# Examination of Independent Prognostic Power of Gene Expressions and Histopathological Imaging Features in Cancer

**DOI:** 10.3390/cancers11030361

**Published:** 2019-03-13

**Authors:** Tingyan Zhong, Mengyun Wu, Shuangge Ma

**Affiliations:** 1 SJTU-Yale Joint Center for Biostatistics, Department of Bioinformatics and Biostatistics, School of Life Sciences and Biotechnology, Shanghai Jiao Tong University, Shanghai 200240, China; tyzhong@sjtu.edu.cn; 2 School of Statistics and Management, Shanghai University of Finance and Economics, Shanghai 200433, China; 3 Department of Biostatistics, Yale University, New Haven, CT 06520, USA

**Keywords:** cancer prognosis, independent prognostic power, omics profiles, histopathological imaging features

## Abstract

Cancer prognosis is of essential interest, and extensive research has been conducted searching for biomarkers with prognostic power. Recent studies have shown that both omics profiles and histopathological imaging features have prognostic power. There are also studies exploring integrating the two types of measurements for prognosis modeling. However, there is a lack of study rigorously examining whether omics measurements have independent prognostic power conditional on histopathological imaging features, and vice versa. In this article, we adopt a rigorous statistical testing framework and test whether an individual gene expression measurement can improve prognosis modeling conditional on high-dimensional imaging features, and a parallel analysis is conducted reversing the roles of gene expressions and imaging features. In the analysis of The Cancer Genome Atlas (TCGA) lung adenocarcinoma and liver hepatocellular carcinoma data, it is found that multiple individual genes, conditional on imaging features, can lead to significant improvement in prognosis modeling; however, individual imaging features, conditional on gene expressions, only offer limited prognostic power. Being among the first to examine the independent prognostic power, this study may assist better understanding the “connectedness” between omics profiles and histopathological imaging features and provide important insights for data integration in cancer modeling.

## 1. Introduction

In cancer research and practice, prognosis is of essential interest. Extensive statistical modeling has been conducted, and yet there is still much room for additional research [1,2]. Multiple families of biomarkers have been used in cancer prognosis modeling. In the past decades, with the development of profiling techniques, omics data have been extensively used and shown to be effective. For example, in [3], a signature composed of 21 gene expressions is used for modeling breast cancer prognosis. In [4], hsa-mir-155 and hsa-let-7a-2 microRNAs are found as prognostic for lung cancer. In [5], the prognostic power of methylated RASSF1A and/or APC serum DNA for breast cancer is identified. Such findings are biologically highly sensible as cancer is a genetic disease and the development and progression of cancer are strongly affected by molecular changes.

Imaging techniques have been extensively used in cancer practice. In particular, for definitive diagnosis, biopsies are usually ordered, and pathologists review the slides of representative sections of tissues. The histopathological imaging data generated in this process directly reflect the histological organization and morphological characteristics of tumor cells and their surrounding tumor microenvironment. More recently, beyond diagnosis, histopathological imaging data have also been used for modeling other cancer outcomes/phenotypes. For example, Harpole et al. [6] showed that the levels of tumor cell dedifferentiation are associated with survival outcomes. Automated histopathological imaging analysis systems have been shown to be effective in the prognostic determination of various malignancies, including breast cancer [7], neuroblastoma [8], lymphoma [9], nonsmall cell lung cancer [10], precancerous lesions in the esophagus [11], and others. 

*Omics profiles and histopathological images are biologically connected and contain overlapping information*. In particular, properties of tumors and their microenvironment, as described in histopathological images, are highly regulated by molecular changes. Multiple statistical modelings have been conducted on their interconnections. For example, Yu et al. [10] found the correlation of quantitative histopathological features with TP53 mutation in lung adenocarcinoma. Cooper et al. [12] demonstrated that PDGFRA, EGFR, and MDM2 amplifications are associated with imaging features such as greater minor axis length, area, and circularity in glioblastoma. 

*On the other hand, it is also possible that omics profiles and histopathological images may contain independent information*. Tissues used to generate omics measurements are usually heterogeneous and mixtures of different components, making the observed omics measurements represent an aggregation of distinct cells [12]. Histopathological images, with extremely high spatial resolutions, also contain information on tissue relationships and characteristics of the spatial organizations of tumor cells. Features of histopathological images, beyond regulated by molecular changes, may also be affected by personal immune system, microenvironment, and other factors. There are a few recent studies integrating omics measurements and imaging features and showing that such a data integration can lead to improved prognosis modeling for breast cancer [13], brain tumor [14], and nonsmall cell lung cancer [15]. Such studies seem to suggest that there is independent information in omics and histopathological imaging data.

Our literature review suggests that there is a lack of study rigorously quantifying whether omics profiles contain independent information, conditional on histopathological images, on cancer prognosis, and vice versa. It is noted that the aforementioned and other data integration studies do not suggest which type of measurement has independent information. In addition, they are mostly estimation-based and do not have a rigorous statistical inference framework. Some studies rely on prescreening to accommodate high data dimensionality and can be statistically limited. This study is conducted to directly tackle these problems. It can advance from the existing literature in multiple aspects. Specifically, it delivers an analysis pipeline which has a rigorous statistical inference framework and can show whether individuals of a type of measurement have additional prognostic power conditional on the other type of measurement. It can provide solid evidence on whether data integration is needed in modeling and clinical practice. In addition, data analysis may also provide further insights into two deadly cancers.

## 2. Data

The Cancer Genome Atlas (TCGA) is one of the largest and most comprehensive cancer projects, and is organized by the National Cancer Institute (NCI) and National Human Genome Research Institute (NHGRI) [16]. It has published high quality omics data and histopathological images for 33 types of cancer. In this study, we analyze data on lung adenocarcinoma (LUAD) and liver hepatocellular carcinoma (LIHC), both of which have high mortality rates and pose increasing public concerns. They also have relatively larger sample sizes, which is critical for making reliable findings. The proposed analysis can be extended to other cancer types straightforwardly. For omics measurements, we consider gene expressions, which have a central role in cancer prognosis modeling. The response of interest is overall survival time, which is right censored. It is noted that other types of omics measurements, for example protein expressions and microRNAs, and other prognosis outcomes can be analyzed in a similar manner.

### 2.1. mRNA Gene Expression Measurements

We download the mRNA gene expression data from cBioPortal (http://cbioportal.org), which have been measured using the Illumina Hiseq2000 RNA Sequencing Version 2 analysis platform, and processed and normalized using the RSEM software. We refer to the literature [17,18] and TCGA website for more detailed information on data collection and processing. Data are available on 25,031 gene expression measurements. As the number of cancer-related genes is not expected to be large, we conduct a simple prescreening to reduce the dimensionality and also improve the stability of estimation. Specifically, the top 5000 genes with the largest variances are selected for downstream analysis.

### 2.2. Histopathological Imaging Features

**Figure 1:**
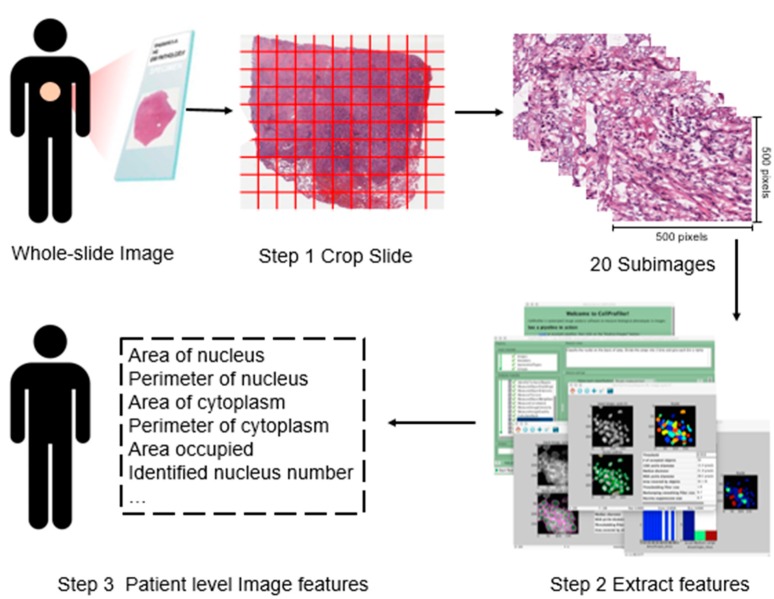
Flowchart of extracting imaging features. Step 1: whole-slide histopathology images are cropped into small subimages of 500 × 500 pixels, and 20 subimages are then randomly selected. Step 2: Imaging features are extracted using CellProfiler [19] for each subimage. Step 3: For each patient, features are averaged.

We download the whole-slide histopathology images directly from the TCGA website (https://portal.gdc.cancer.gov), which are in the svs format. These tissue slides are formalin-fixed, paraffin-embedded slides with which the cell morphology is well-preserved and thus appropriate for image feature recognition. They are captured at 20× or 40× magnification by the Aperio medical scanner. To extract imaging features, we conduct the following three-step preprocessing. The overall flowchart is provided in Figure 1.

In the first step, we process the downloaded svs files using the Openslide Python library [20]. Specifically, to make data in an appropriate format for morphological feature extraction, each svs image is cropped into small subimages of 500 × 500 pixels, and each subimage is then saved in the tiff format. Among them, subimages that contain mostly (>50%) background white space are filtered out. From the remaining subimages, twenty representative ones are randomly selected for each svs image to avoid potential subjective bias and reduce computational cost [21].

In the second step, we use CellProfiler [19] to extract features from each subimage. CellProfiler is an open source cell image analysis platform developed by the Broad Institute. Specifically, image colors are separated based on hematoxylin staining and eosin staining, and converted to grayscale to extract regional features, such as cell and tissue texture and granularity. These are “classic” image processing features, which have been examined in a large number of imaging studies. It is noted that they are not expected to be specific histopathological features, and cannot be recognized by pathologist. More specifically, texture describes a set of metrics calculated in CellProfiler to quantify the perceived texture of histopathological images, and includes information on the spatial arrangement of color or intensities in images. Granularity describes the size of how well the structure element fits in images. Then, cell nuclei are identified and segmented to collect the cell-level features, such as cell size, shape, distribution, and texture of nuclei. Other cell features such as regional occupation, area fraction, and neighboring architecture are also captured. The above process generates 832 features for each subimage. A further screening is conducted to exclude irrelevant features such as file size and execution information. Finally, for each subimage, a total of 772 features are available for analysis.

In the third step, for each slide, the average feature values over twenty representative subimages are computed. When a subject has multiple slides, the average values over multiple slides are further computed for downstream analysis.

The above preprocessing is applied to both LUAD and LIHC data. There are missing values in imaging feature data. Subjects with more than 25% missing imaging features are excluded. The remaining missing values are imputed using sample medians.

**Remark** **1.** We adopt CellProfiler to extract imaging features, which is a popular choice in recent literature. The feature names are automatically provided by CellProfiler. The extracted features represent objective attributes of histopathological images, including the area and perimeter of nucleus and cytoplasm, mean and standard deviation of these measures, and other general image attributes. For each patient, multiple slides and subimages may be available. To extract as much information as possible, we consider multiple slides and subimages simultaneously, and the average values are used to summarize information. A closer examination suggests that they are not explicitly associated with specific histopathological findings, such as cellularity, atypia, anaplasia, nuclear pleomorphism, and some others. However, these attributes have been shown to be associated with pathological stages [7,10] and prognosis [21], and also been used in multiple existing cancer modeling studies [13,15]. For example, Yu et al. [22] show that some of these features are associated with specific subtypes of lung cancer. In this study, our goal is to rigorously quantify independent information in omics profiles and histopathological images for cancer prognosis, as opposed to focusing on specific interpretation of imaging features. As such, these features are sensible for our analysis, although they may not have simple, explicit biological interpretations.

### 2.3. Available Data

After data matching, we obtain records on 316 and 358 subjects for LUAD and LIHC, respectively, with 5000 gene expression measurements, 772 imaging features, and survival time. For LUAD, there are 103 deaths during follow-up, with survival times ranging from 0 to 214.77 months (median 6.03 months). For LIHC, there are 121 deaths during follow-up, with survival times ranging from 0 to 120.73 months (median 19.25 months). Summary information of the analyzed subjects is provided in Table 1.

## 3. Methods

For describing cancer survival, we use the accelerated failure time (AFT) model. This model has been extensively adopted in cancer studies with high-dimensional variables because of its lucid interpretations and low computational cost [23]. For each gene expression, we consider the prognosis model with its effect along with all imaging features. A statistically rigorous test is then conducted on the gene expression’s effect, which can suggest whether this particular gene has independent information for prognosis conditional on the imaging features. Then a parallel analysis is conducted, reversing the roles of gene expressions and imaging features. With a special emphasis on omics and imaging features, clinical factors are not included in the prognosis models.

Consider *n* independent subjects. For the *i*th subject, denote $x_{i}=\left(x_{i1},\;x_{x2},\;\ldots ,\;x_{ip}\right)$ and $z_{i}=\left(z_{i1},\;z_{i2},\;\ldots ,\;z_{iq}\right)$ as the *p*- and *q*-dimensional vectors of gene expressions and imaging features, $T_{i}$ and $C_{i}$ as the logarithm of the event and censoring times. With right censoring, we observe $\left(y_{i}=\min\left(T_{i},C_{i}\right),\delta_{i}=I\left(T_{i}\leq C_{i}\right),x_{i},z_{i}\right)$.

First consider the analysis with one gene expression and all imaging features. The analysis with one imaging feature and all gene expressions can be conducted in a similar manner and will not be reiterated. For the *j*th gene expression, consider the following AFT model:

(1) $$T_{i}=\alpha +x_{ij}\beta_{j}+z_{i}\theta_{j}+\varepsilon_{i}$$

where α is the intercept, $\beta_{j}$ and $\theta_{j}={\left(\theta_{j1},\ldots ,\theta_{jq}\right)}^{\prime }$ are the unknown coefficients for the *j*th gene expression and all imaging features, and $\varepsilon_{i}$ is the random error. A statistical test

$$H_{0}:\;\beta_{j}=0\;\mathrm{vs}.\;H_{1}:\;\beta_{j}\neq 0$$

can reveal whether $x_{ij}$ is independently associated with $T_{i}$ given $z_{i}$. Here, loosely speaking, a smaller *p* value can indicate a stronger association/more prognostic power. The analysis is challenging with the high dimensionality of imaging features, which makes the “standard” estimation and inference techniques not applicable. To tackle this problem, we consider a high-dimensional inference approach [24] recently developed under a related but simpler context. Specifically, for estimation, the weighted least squares approach is adopted. Assume that data have been sorted according to $y_{i}$’s from the smallest to the largest. Then we have the Kaplan–Meier weights defined as

$$w_{1}=\frac{\delta_{1}}{n},\;w_{i}=\frac{y_{i}}{n-i+1}\prod_{j=1}^{i-1}{\left(\frac{n-j}{n-j+1}\right)}^{\delta_{j}},i=2,\ldots ,n,$$

where a further normalization is conducted to make $\sum_{i=1}^{n}w_{i}=n$. Let $W=\mathrm{diag}\left\{w_{1}\;,w_{2},w_{3},\ldots ,w_{n}\right\}$, $x_{\cdot j}$, $z_{\cdot k}$, and y denote the vectors composed of $x_{ij}$’s, $z_{ik}$’s, and $y_{i}$’s, which are weighted normalized such that $1^{\prime }Wx_{\cdot j}=0,\;1^{\prime }Wz_{\cdot k}=0,\;\mathrm{and}\;1\prime Wy=0$, and $Z=\left(z_{\cdot 1},\ldots ,z_{\cdot q}\right)$. With the high data dimensionality, regularized estimation is needed. In addition, not all imaging features are expected to be associated with survival, posing a variable selection problem. Consider the weighted penalized estimation:

(2) $$\{\begin{array}{c} \left({\tilde{\beta }}_{j}^{*},{\tilde{\theta }}_{j}\right)={\mathrm{argmin}}_{\beta_{j},\theta \in R^{1+q}}\left\{\frac{1}{2n}\|W^{\frac{1}{2}}{\left(y-x_{\cdot j}\beta_{j}-Z\theta_{j}\right)\|}_{2}^{2}+\lambda_{0}\sum_{k=1}^{q}\left|\theta_{jk}\right|\right\}\\ {\tilde{b}}_{j}={\mathrm{argmin}}_{b_{j}\in R^{q}}\left\{\frac{1}{2n}\|W^{\frac{1}{2}}{\left(x_{\cdot j}-Zb_{j}\right)\|}_{2}^{2}+\lambda_{1}\sum_{k=1}^{q}\left|b_{jk}\right|\right\} \end{array},$$

where ${\left\|\cdot \right\|}_{2}$ denotes the L_2_ norm, $b_{j}={\left(b_{j1},\ldots ,b_{jq}\right)}^{\prime }$ is the unknown coefficient vector, and $\lambda_{0}$, $\lambda_{1}$ > 0 are data-dependent tuning parameters for Lasso penalties. 

With ${\tilde{\theta }}_{j}$ and ${\tilde{b}}_{j}$, the final estimate ${\tilde{\beta }}_{j}$ of $\beta_{j}$ is defined as

(3) $${\tilde{\beta }}_{j}={\left({\tilde{x}}_{.j}{}^{\prime }Wx_{.j}\right)}^{-1}{\tilde{x}}_{.j}{}^{\prime }W\left(y-Z{\tilde{\theta }}_{j}\right),$$

where ${\tilde{x}}_{.j}=x_{\cdot j}-Z{\tilde{b}}_{j}$. It has been shown in [24] that $\sqrt{n}\left({\tilde{\beta }}_{j}-\beta_{j}\right)$ is asymptotically normal with variance defined as

$$\tilde{\Sigma }\left({\tilde{\beta }}_{j}\right)=\frac{1}{n}{\left({\tilde{x}}_{.j}{}^{\prime }Wx_{.j}\right)}^{-1}\tilde{\Sigma_{1}}{\left(x_{.j}{}^{\prime }W{\tilde{x}}_{.j}\right)}^{-1},$$

where $\tilde{\Sigma_{1}}$ is the sample variance based on ${\tilde{x}}_{ij}(y_{i}-x_{ij}{\tilde{\beta }}_{j}+z_{i}{\tilde{\theta }}_{j})$. With this asymptotic normal distribution, the test statistic for $H_{0}:{\;\beta }_{j}=0$ can be defined as

$${\tilde{\beta }}_{j}/\sqrt{\tilde{\Sigma }\left({\tilde{\beta }}_{j}\right)/n},$$

which follows Student’s T distribution. The unadjusted *p* value can then be obtained. When all gene expressions are considered together, to accommodate multiple comparisons, *p* values are adjusted using the voxel-level false discovery rate approach [25].

An advantage of the above analysis is that it can be realized via simple coding. The most challenging step is the estimation in (2), which can be realized using the R function *glmnet*. The tuning parameters $\lambda_{0}$ and $\lambda_{1}$ are selected using the EBIC approach [26]. To facilitate data analysis in and beyond this study, we have developed R code implementing the proposed approach. To illustrate its usage, we have also provided an example R file with the LUAD data. The code and data are publicly available at http://www.github.com/shuanggema/TestLDHD as well as in Supplementary Materials.

**Remark** **2.** The effectiveness of the AFT model for cancer prognosis modeling has been well tested. Penalization has been shown effective for screening out irrelevant variables and accommodating high dimensionality. As shown in [24], the estimation (2) can effectively “single out” the effect of the one gene expression. It is noted that, as the gene expression effect is of particular interest, its coefficient is not subject to penalization in estimation. A “byproduct” of penalized estimation is that imaging features associated with prognosis, conditional on the one gene expression effect, are identified, which may assist understanding the associations between imaging features and prognosis as well as the associations between imaging features and gene expressions. The statistical distribution result for the test statistic has been rigorously proved in [24]. The T distribution makes inference very easy.

## 4. Results

### 4.1. Identification of Gene Expressions with Independent Prognostic Power Conditional on Imaging Features

**Figure 2:**
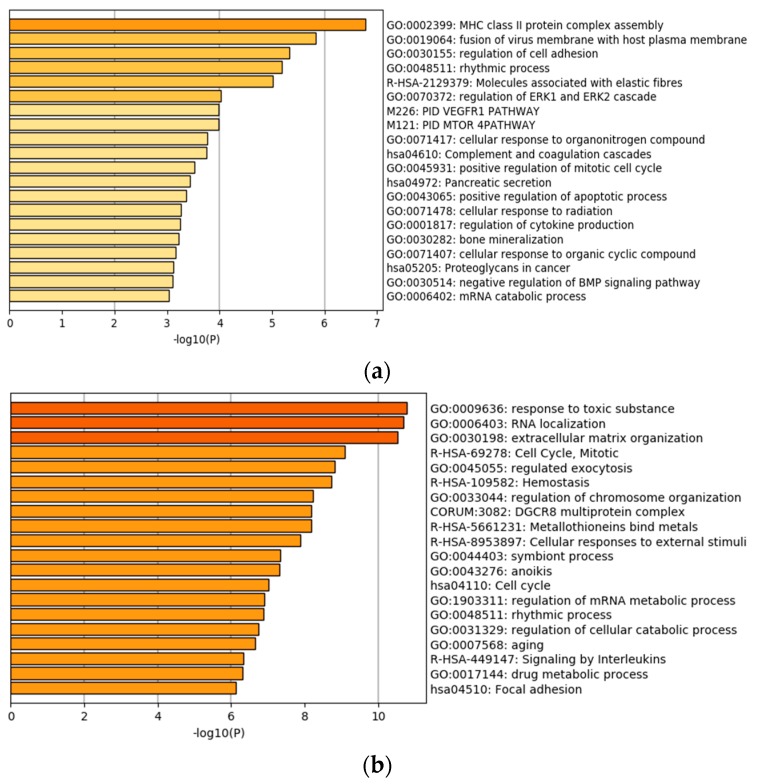
Gene ontology (GO) and pathway enrichment analysis of the identified genes. (**a**) lung adenocarcinoma (LUAD), (**b**) liver hepatocellular carcinoma (LIHC).

We first apply the analysis approach described above to identify individual gene expressions that have independent prognostic power conditional on the high dimensional histopathological imaging features. With significance level 0.05 as cutoff, 85 genes are identified as significantly associated with prognosis for LUAD. Detailed estimation results are provided in the Table S1. A quick literature search of PubMed suggests that many of the findings have sound biological basis. For top ten representative genes, we provide brief information and references on their associations with lung cancer prognosis in Table 2. For LIHC, we identify 386 genes as significantly associated with prognosis conditional on imaging features. Again, it is found that many of those genes have established evidences of being associated with liver cancer prognosis. Brief information on the representative genes is provided in Table 3. The solid evidences from existing studies provide support to the validity of analysis. To more comprehensively comprehend the identified genes, we examine the genes’ functional and biological connections by conducting Gene Ontology (GO) and KEGG pathway enrichment analysis. The processes with the smallest *p* values are presented in Figure 2. It is observed that both LUAD and LIHC have enriched processes associated with cell adhesion. This may result from high-dimensional imaging features capturing more information on cell interaction. The identified genes are also enriched in traditional cancer hallmarks, such as the positive regulation of mitotic cell cycle, proteoglycans in cancer, and extracellular matrix regulation. It is interesting that some immune response-related pathways, such as MHC assembly, are also identified, considering that there are promising developments in immunotherapy for cancer treatment recently.

It is also noted that the proposed analysis takes an angle different from the literature. As such, there are also new findings. For example, the top ranked genes (with the smallest *p* values) also include DCAF6 and LITAF for LUAD and IARS and LRPPRC for LIHC. Gene DCAF6 is a transcriptional cofactor that enhances androgen receptor (AR)-mediated transcriptional activity. Chen et al. [27] have found that the expression of DCAF6 is upregulated in prostate cancer patient and may be a candidate tumor promoter for prostate cancer, indicating its important role in cancer etiology. For gene LITAF, Zhou et al. [28] have established the regulatory axis of AMPK–LITAF–TNFSF15, where AMPK activation upregulates the transcription of LITAF and consequently upregulates the expression of TNFSF15. TNFSF15 inhibits the growth of prostate cancer cells and bovine aortic endothelial cells in vitro, supporting that LITAF may function as a tumor suppressor. Gene IARS is the coding gene of isoleucyl-tRNA synthetase (ARSs). ARSs has been shown to catalyze the amino acylation of tRNAs by their cognate amino acid, linking amino acids with the correct nucleotide triplets and ensuring the correct transformation of the genetic code to the protein level. Kopajtich et al. [29] have reported that biallelic IARS mutations can cause infantile hepatopathy, suggesting its potential association with liver function. LRPPRC is another interesting gene, which regulates the expression of all mitochondrial DNA-encoded mRNAs, and thus has important contributions to mitochondrial function. Tian et al. [30] have examined the expression of LRPPRC in six types of cancer and observed that LRPPRC plays an important role in tumorigenesis through the resistance to apoptosis and high invasive activity. Other newly identified genes, such as FLS353 [31], IPO7 [32], PDS5A [33], and MPP-2 [34], have also been demonstrated to be related to tumorigenesis. This brief literature search suggests that the implications of these new findings in lung and liver cancer prognosis have not been well established, however, they have been observed to have important contributions to cancer etiology. The new findings are also not surprising. It is conceptually sensible that the strongest “signals” are perhaps reflected in both omics profiles and histopathological images. The proposed analysis seeks for additional “signals” in gene expressions that are not reflected in images. As such, the findings may complement those in the literature.

**Figure 3:**
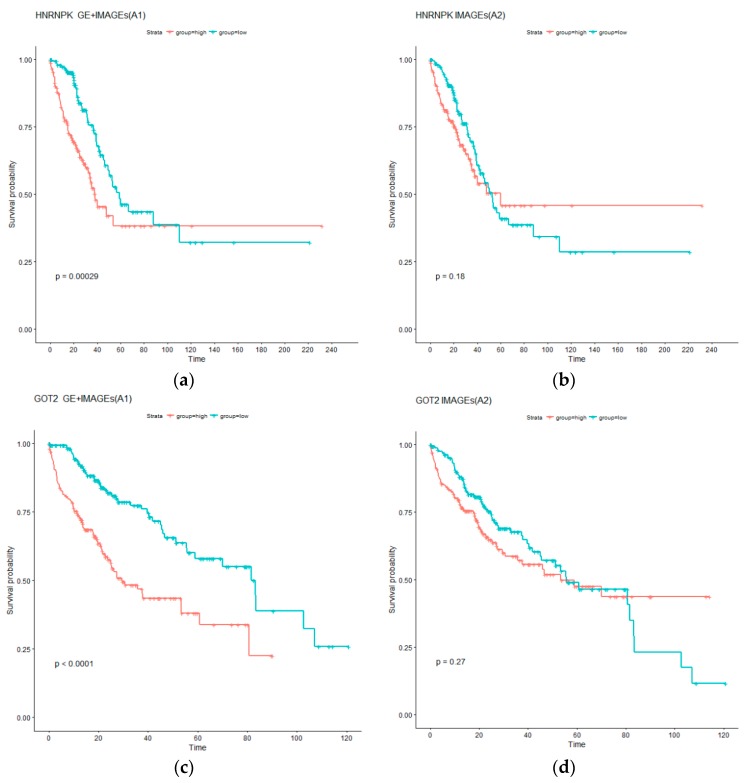
Kaplan–Meier (KM) curves for low (blue) and high (red) risk groups under models A1 and A2. (**a**,**b**) for LUAD: Gene HNRNPK as well as selected imaging features (A1), and only selected imaging features (A2). (**c**,**d**) for LIHC: Gene GOT2 as well as selected imaging features (A1), and only selected imaging features (A2). *p* values are computed from log rank tests.

Analysis is further conducted to better comprehend the additional prognostic information in gene expressions. Specifically, for each identified gene expression, two AFT models are considered. The first model, referred to as A1, contains the single gene expression as well as selected imaging features; in contrast, the second model, referred to as A2, contains only the selected imaging features. Comparing the two models can straightforwardly describe the contribution of the gene expression to prognosis. To facilitate the comparison, subjects are classified into low and high risk groups with equal sizes based on the survival times predicted using A1 and A2, and the log rank test is conducted to compare survival of the two groups. *p* values from the log rank tests are provided in the Table S1. For LUAD, it is observed that 56 out of the 85 A1 type models can effectively separate the high and low risk groups (with the adjusted log rank test *p* values < 0.05); In contrast, 47 of the A2 type models have significant adjusted log rank *p* values. In addition, 51 A1 type models have *p* values smaller than their A2 counterparts. In the analysis of LIHC data, the numbers of significant adjusted *p* values for the A1 and A2 type of models are 237 and 66, respectively. In addition, 286 genes have *p* values smaller under the A1 type models than their A2 counterparts. These results suggest that gene expressions can significantly improve prognosis modeling beyond histopathological imaging features. For a more intuitive presentation, we present the Kaplan–Meier curves for two representative genes in Figure 3. Specifically, we show the survival functions of the low and high risk groups under the A1 and A2 models. The A1 models have a much clearer and more significant separation. Such results suggest that, for clinical practice, it may be necessary to integrate gene expression data beyond histopathological imaging data.

### 4.2. Identification of Imaging Features with Independent Prognostic Power Conditional on Gene Expressions

Analysis parallel to the above is conducted, reversing the roles of gene expressions and imaging features. With significance level 0.05, the identified imaging features along with their *p* values are shown in Table 4. Detailed estimation results are provided in the Table S1. In the LUAD data analysis, 11 imaging features are found as significantly associated with prognosis conditional on the selected gene expressions. Among them, six are texture related, which is consistent with the literature [21]. In the analysis of LIHC data, nine imaging features are found as significantly associated with prognosis conditional on gene expressions, among which eight belong to the morphological category of nuclei texture. There is one notable difference between gene expression and histopathological imaging measurements. With extensive functional studies accumulated over years, the biological functions of many genes are known; In contrast, the biological interpretations of imaging features mostly remain unclear. As such, interpretations as in Table 2; Table 3 cannot be pursued.

**Figure 4:**
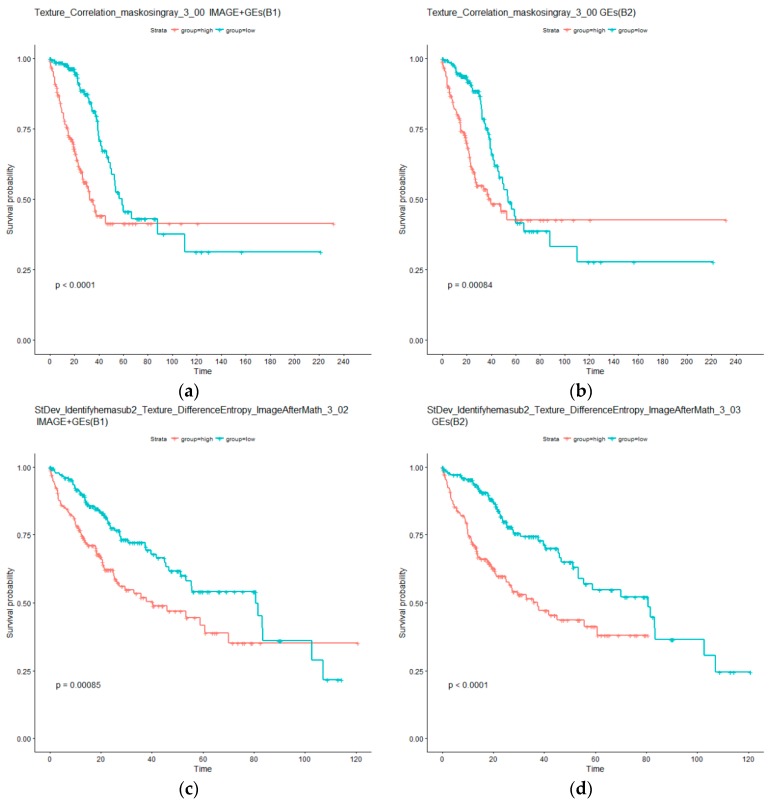
KM curves for low (blue) and high (red) risk groups under models B1 and B2. (**a**,**b**) for LUAD: Imaging feature Texture_Correlation_maskosingray_3_00 as well as selected gene expressions (B1), and only selected gene expressions (B2). (**c**,**d**) for LIHC: Imaging feature StDev_Identifyhemasub2_Texture_DifferenceEntropy_ImageAfterMath_3_00 as well as selected gene expressions (B1), and only selected gene expressions (B2). *p* values are computed from log rank tests.

As in the above section, additional analysis is conducted. Specifically, two types of AFT models are considered. The first type, referred to as B1, is based on an individual imaging feature and selected gene expressions; whereas the second type, referred to as B2, is only based on selected gene expressions. Detailed results are provided in the Table S1. For LUAD, it is observed that ten out of the 11 B1 type models can effectively separate the high and low risk groups, whereas 11 of the B2 type models have significant adjusted log rank *p* values. For the identified imaging feature Granularity_15_ImageAfterMath, the B1 model is not significant, however, the corresponding B2 model is significant. This can be explained by the larger degrees of freedom of the B1 model and possible correlation between the two types of measurements. It is also found that six imaging features have *p* values smaller under the B1 type models than their B2 counterparts. In the LIHC data analysis, the overall findings are similar. Specifically, all tests under both B1 and B2 types of models have adjusted *p* values smaller than 0.05. All tests under B1 have *p* values larger than those under B2. Again, this can be possibly explained by the larger numbers of parameters under B1 and correlations. The KM curves for two representative imaging features are examined in Figure 4. The differences between the left and right panels are much less distinct compared to Figure 3. Overall, the analysis suggests that, conditional on the presence of gene expression measurements, histopathological imaging features have limited independent prognostic power.

## 5. Discussion

In the literature, the separate and integrated analyses of omics and histopathological imaging data have been conducted. This study has taken a different perspective and tried to answer the fundamental question of whether a type of measurement has independent prognostic power conditional on the collective effect of the other type. A rigorous statistical testing approach, developed under simpler settings, has been adopted and extended. In the analysis of TCGA data on two cancer types, it has been found that, conditional on imaging features, individual gene expressions may still have significant prognostic power; however, conditional on gene expressions, individual imaging features may have limited prognostic power. As such, at least for the analyzed datasets, gene expressions and histopathological imaging features have an irreversible independent relationship in modeling prognosis. Such findings, to the best of our knowledge, are the first in the literature. They may have important implications for cancer practice. Specifically, in cancer clinical practice, gene expression profiling is becoming routine. Our analysis suggests that, for modeling prognosis, when gene expression data are available, clinicians may not need to order histopathological imaging. However, this is not true the other way around. For a more accurate prognosis modeling, with imaging data, clinicians may still want to order gene expression profiling, and use integrated analysis techniques (developed in published and future literature) to integrate gene expression and imaging data. The biological implications of the findings, pertinent to the associations between gene expressions and imaging features, are worth further investigation.

This study can be extended in multiple directions. Specifically, other types of omics measurements, such as DNA mutations, miRNA expressions, methylation, and copy number variations, can be analyzed similarly. It is noted that the findings are not necessarily the same as in this article. In our analysis, we have considered high dimensional imaging features, which may include more information but do not have simple biological interpretations. It is possible to conduct a similar analysis using low dimensional imaging features such as vascular invasion and lymphocyte cells—this is postponed to future research. It is noted that in the literature [55], techniques have already been developed to identify cancer regions, extract such low dimensional imaging features, and use them in analysis. The analysis has been focused on gene expression and imaging data. We acknowledge the importance of clinical, environmental, and other factors in cancer prognosis. These factors are not included in analysis with the following concerns. First, the most important objective of this study is to evaluate the overlapping/independent information in omics and histopathological imaging data. As such, we focus on these two types of measurements. Second, to the best of our knowledge, techniques for making inference with the “one gene expression + low dimensional clinical factors + high dimensional imaging features” model are not available. This analysis can be more complex than that in this article with the significant differences between clinical factors and imaging features. Table 1 has been provided so that researchers can comprehend properties of the TCGA cohorts. Extending the proposed analysis and accommodating clinical factors will be postponed to future research. It is also of interest to conduct similar analysis for other types of cancer outcomes. For a continuous cancer marker, the described analysis technique can be directly applied. New developments will be needed for other types of outcomes/phenotypes. It is also noted that the detailed results can be data and model dependent. It is impossible to conduct analysis with all cancer types and models. However, the “spirit” of the proposed analysis will be broadly applicable. Although with certain limitations, being the first of its kind, this study may still provide important insights into cancer modeling and characteristics of cancer as reflected in omics profiles and histopathological images.

## 6. Conclusions

Omics and histopathological imaging data have been co-collected in cancer practice and analyzed in recent integrated analysis. In this study, we have presented a pipeline for analyzing their independent prognostic power conditional on the other type of data. The adopted statistical inference technique has a solid ground and can be broadly applied. The “asymmetric” finding is interesting, has not been observed in the literature, and has sound interpretations. It is reasonable to expect that the proposed analysis and its downstream integrated analysis will gain popularity fast and have a deep impact on cancer practice.

## Figures and Tables

**Table 1:** Sample characteristics.

Characteristic	LUAD	LIHC
Sample size	316	358
Age at diagnosis: median (range)	66 (39–88)	61 (16–90)
Follow-up: median (range)	6.03 (0–214.77)	19.25 (0–120.73)
Vital status: *n* (%)		
Alive	213 (67.4%)	233 (65.0%)
Deceased	103 (32.6%)	125 (35.0%)
Sex: *n* (%)		
Male	144 (45.6%)	242 (67.6%)
Female	172 (54.4%)	116 (32.4%)
Cancer stage: *n* (%)		
I	180 (57.0%)	166 (46.4%)
II	69 (18.7%)	82 (22.9%)
III	41 (13%)	83 (23.2%)
IV	21 (6.6%)	5 (1.4%)
NA	5 (1.6%)	22 (6.1%)

**Table 2:** Analysis of LUAD data: representative identified genes.

Gene	Evidence	PMID
*HYAL2*	Real-time PCR studies showed that HYAL2 genes were down regulated in non-small cell lung cancer [35].	19140316
*MAPK1IP1L*	MAPK1IP1L gene was found to be related with acquired resistance to MET inhibitors in lung cancer cells [36].	28396363
*HLA-DRA*	Lack of surface class II expression was found to be associated with a specific defect in HLA-DRA induction in non-small cell lung carcinoma cells [37].	8786310
*HNRNPK*	Higher levels of hnRNP mRNAs were found in SCLC as compared to NSCLC. hnRNP K protein localization varied with cellular confluence [38].	12871776
*GPNMB*	Osteoactivin (GPNMB) ectodomain protein was shown to promote growth and invasive behavior of human lung cancer cells [39].	26883195
*BMP2*	Positive correlation was found between gene expressions of two angiogenic factors, VEGF and BMP-2, in lung cancer patients [40].	19324447
*COMMD6*	COMMD9 was demonstrated to promote TFDP1/E2F1 transcriptional activity via interaction with TFDP1 in non-small cell lung cancer [41].	27871936
*HLA-DRB1*	Lung cancer patients in Japan showed an increased frequency of HLA-DRB1*0901 and a decreased frequency of HLA-DRB1*1302 and DRB1*14-related alleles when compared to the other subjects [42].	9808426
*LARP1*	LARP1 post-transcriptionally regulates mTOR and contributes to cancer progression [43].	25531318
*ZAK*	ZAK inhibits human lung cancer cell growth via ERK and JNK activation in an AP-1-dependent manner [44].	20331627

* The star is a sign indicating the location of the mutated allel.

**Table 3:** Analysis of LIHC data: representative identified genes.

Gene	Evidence	PMID
*LAPTM4B*	LAPTM4B is a potential proto-oncogene, whose overexpression is involved in carcinogenesis and progression of HCC [45].	12902989
*CAPZA1*	CAPZA1 expression levels were negatively correlated with the biological characteristics of primary HCC and patient prognosis [46].	28093067
*PLOD2*	PLOD2 expression was identified as a significant, independent factor of poor prognosis for HCC patients [47].	22098155
*STIP1*	STIP1 was upregulated in HCC and associated with poor clinical prognosis [48].	28887036
*IGF1*	Inhibition of IGF-1R tyrosine kinase (IGF-1R-TK) by NVP-AEW541 induces growth inhibition, apoptosis and cell cycle arrest in human HCC cell lines without accompanying cytotoxicity [49].	16530734
*HTATIP2*	HepG2 cells that expressed transgenic HTATIP2 formed more invasive tumors in mice following administration of sorafenib. Sorafenib therapy prolonged recurrence-free survival in patients who expressed lower levels of HTATIP2 compared with higher levels [50].	22922424
*GNAI3*	GNAI3 inhibits tumor cell migration and invasion and is post-transcriptionally regulated by miR-222 in hepatocellular carcinoma [51].	25444921
*XPO1*	Exportin-1 (XPO1, CRM1) mediates the nuclear export of several key growth regulatory and tumor suppressor proteins [52].	25030088
*PLVAP*	PLVAP was identified as a gene specifically expressed in vascular endothelial cells of HCC but not in non-tumorous liver tissues [53].	25376302
*EPAS1*	HIF-2alpha/EPAS1 expression may play an important role in tumor progression and prognosis of HCC [54].	17589895

**Table 4:** Identified histopathological features.

LUAD		LIHC	
Feature Name	Adjusted *p* Value	Feature Name	Adjusted *p* Value
Mean_Identifyeosinprimarycytoplasm_Texture_Correlation_maskosingray_3_00	1.16 × 10^−4^	StDev_Identifyhemasub2_Texture_DifferenceEntropy_ImageAfterMath_3_02	9.46 × 10^−6^
Median_Identifyeosinprimarycytoplasm_Texture_Correlation_maskosingray_3_00	1.59 × 10^−4^	StDev_Identifyhemasub2_Texture_SumEntropy_ImageAfterMath_3_00	9.46 × 10^−6^
StDev_Identifyeosinprimarycytoplasm_Texture_Correlation_maskosingray_3_00	1.59 × 10^−4^	StDev_Identifyhemasub2_Texture_SumEntropy_ImageAfterMath_3_02	1.85 × 10^−5^
StDev_Identifyhemasub2_AreaShape_Orientation	1.59 × 10^−4^	StDev_Identifyhemasub2_Texture_DifferenceEntropy_ImageAfterMath_3_00	2.92 × 10^−5^
StDev_Identifyhemasub2_AreaShape_Zernike_6_6	1.05 × 10^−4^	StDev_Identifyhemasub2_Texture_SumEntropy_ImageAfterMath_3_01	3.64 × 10^−5^
StDev_Identifyhemasub2_AreaShape_Zernike_9_1	1.59 × 10^−4^	StDev_Identifyhemasub2_Texture_DifferenceEntropy_ImageAfterMath_3_01	4.08 × 10^−5^
StDev_Identifyhemasub2_AreaShape_Zernike_9_9	1.59 × 10^−4^	StDev_Identifyhemasub2_Texture_DifferenceEntropy_ImageAfterMath_3_03	4.82 × 10^−5^
StDev_Identifyhemasub2_Texture_DifferenceEntropy_ImageAfterMath_3_03	1.64 × 10^−4^	StDev_Identifyhemasub2_Texture_SumEntropy_ImageAfterMath_3_03	9.24 × 10^−5^
StDev_Identifyhemasub2_Texture_SumEntropy_ImageAfterMath_3_01	1.64 × 10^−4^	Granularity_2_ImageAfterMath	1.07 × 10^−4^
Texture_Correlation_maskosingray_3_00	1.59 × 10^−4^		
Granularity_15_ImageAfterMath	7.66 × 10^−4^		

## Supplementary Materials

The following are available online at https://www.mdpi.com/2072-6694/11/3/361/s1, Table S1: Detailed estimation results referred to in Section 4, TestLDHD: R code and example data.
